# Beyond Oligodendroglioma: An Integrated Diagnostic Approach to CNS Tumors with Oligodendroglioma-like Morphology, with a Focus on Morphological Pitfalls, Immunoprofiles, and Molecular Signatures

**DOI:** 10.3390/ijms27167163

**Published:** 2026-08-11

**Authors:** Giulio Attanasio, Rosario Caltabiano, Francesca Amato, Giuseppe Maria Vincenzo Barbagallo, Francesco Certo, Durmus Ayan, Valeria Barresi, Giuseppe Broggi

**Affiliations:** 1 Pathology Unit, Cannizzaro Hospital, 95126 Catania, Italy; attanasiogiulio@icloud.com; 2 Department of Medical and Surgical Sciences and Advanced Technologies “G.F. Ingrassia”, Anatomic Pathology, University of Catania, 95123 Catania, Italy; rosario.caltabiano@unict.it (R.C.); francescaamato277@gmail.com (F.A.); 3 Department of Medical and Surgical Sciences and Advanced Technologies “G.F. Ingrassia”, Neurological Surgery, Policlinico “G. Rodolico-San Marco” University Hospital, University of Catania, 95123 Catania, Italy; gbarbagallo@unict.it (G.M.V.B.); francesco.certo@unict.it (F.C.); 4 Faculty of Medicine, Medical Biochemistry, Nigde Omer Halisdemir University, 51200 Nigde, Türkiye; durmusayan@hotmail.com; 5 Dr. Abdurrahman Yurtaslan Ankara Oncology Training and Research Hospital, 06200 Ankara, Türkiye; 6 Department of Diagnostics and Public Health, University of Verona, 37134 Verona, Italy; valeria.barresi@istituto-besta.it; 7 Pathology Unit, Fondazione IRCCS Istituto Neurologico Carlo Besta, 20133 Milan, Italy

**Keywords:** oligodendroglioma, oligodendrocyte-like cells, clear-cell CNS tumors, integrated diagnosis, molecular neuropathology, DNA methylation profiling, glioneuronal tumors

## Abstract

Oligodendroglioma-like morphology, classically recognized by round nuclei, optically clear cytoplasm, and perinuclear halos, is one of the most familiar patterns in neuropathology but also one of the most diagnostically misleading. Although historically associated with oligodendroglioma, this phenotype is now recognized across a wide spectrum of neoplastic and non-neoplastic central nervous system lesions, including adult-type diffuse gliomas, ependymal tumors, pediatric-type low-grade gliomas, glioneuronal and neurocytic tumors, metastatic clear-cell neoplasms, demyelinating disease, and subacute infarcts. The 2021 WHO Classification of CNS Tumors has consolidated a diagnostic framework in which histology remains indispensable, but no longer sufficient, for tumor classification. In this setting, oligodendroglioma-like morphology should be interpreted as a morphological clue that prompts a differential diagnosis and guides ancillary testing rather than as a definitive diagnostic category. This review provides a practical integrated approach to CNS tumors and tumor-like lesions with oligodendroglioma-like or clear-cell morphology. We summarize the major diagnostic mimics of oligodendroglioma, highlighting first-line immunohistochemical panels, and proposing a tiered molecular workflow to identify the conditions in which genome-wide DNA methylation profiling becomes essential. Particular emphasis is placed on clinico-pathological correlation and on red flags that should prompt reconsideration of a conventional oligodendroglioma diagnosis.

## 1. Introduction

Over the past two decades, the classification of central nervous system tumors has evolved from a predominantly morphology-centered system to an integrated morphomolecular framework. Histology remains the indispensable first diagnostic tool of neuropathology, but it can no longer be considered sufficient to define tumor identity, biological behavior, prognosis, or therapeutic vulnerability in many CNS entities [1,2]. Successive WHO classifications have progressively incorporated molecular criteria, culminating in the 2021 edition, in which selected genetic and epigenetic alterations are mandatory for the definition of several diagnostic categories [1].

This conceptual transition is particularly relevant for lesions showing oligodendroglioma-like or clear-cell morphology. The classic “fried egg” appearance, with round nuclei and perinuclear halos, was historically regarded as highly suggestive of oligodendroglioma. It is now clear that the same appearance may be encountered in diffuse gliomas, ependymomas, glioneuronal and neurocytic tumors, embryonal or methylation-defined entities, metastatic tumors, and non-neoplastic mimics such as demyelinating lesions or subacute infarcts [1,2,3,4,5,6,7,8,9,10,11,12,13,14,15,16,17,18,19,20,21,22,23,24,25,26,27,28,29,30,31,32,33,34,35,36,37,38,39]. The potential diagnostic pitfall lies in the fact that the morphology may appear deceptively uniform, whereas the underlying biology may be radically different.

Within contemporary practice, the pathologist’s task is therefore not merely to recognize an oligodendroglioma-like pattern, but to determine which biological entity is producing it. Morphology functions as the entry point of a diagnostic algorithm. It should trigger an appropriately prioritized sequence of clinico-radiological correlation, immunohistochemistry, targeted molecular testing, and, in selected cases, genome-wide DNA methylation profiling [1,2,3,40,41,42].

This review proposes a structured and practical approach to CNS lesions with oligodendroglioma-like or clear-cell morphology. The main goal is to offer a diagnostic workflow that can be adapted to centers with variable access to advanced molecular diagnostics.

## 2. Oligodendroglioma-like Morphology: A Pattern, Not a Diagnosis

Oligodendroglioma-like morphology does not denote a specific nosological entity. It describes a recurring microscopic phenotype characterized by relatively uniform round cells, optically clear cytoplasm, perinuclear halos, and, in some cases, a delicate capillary network. The cytoplasmic clearing often reflects fixation-related retraction rather than a lineage-specific feature. Consequently, the same appearance may occur in glial, ependymal, neuronal, glioneuronal, meningeal, metastatic, inflammatory, and ischemic processes.

Recognition of this convergence is critical. A true oligodendroglioma, IDH-mutant and 1p/19q-codeleted, has a distinct prognosis and therapeutic sensitivity. By contrast, an IDH-wildtype glioblastoma with small cell or oligodendroglioma-like morphology, a supratentorial ZFTA-fused ependymoma, a diffuse glioneuronal tumor with oligodendroglioma-like features and nuclear clusters, a metastatic renal cell carcinoma, or a tumefactive demyelinating lesion may all enter the differential diagnosis at low power. These entities require different reporting language, clinical management, and prognostic counselling (Table 1).

The practical implication is that the phrase “oligodendroglioma-like” should be used as a morphological descriptor and diagnostic prompt and that it should not be allowed to function as a substitute for integrated classification.

## 3. Diagnostic Spectrum

### 3.1. Adult-Type Diffuse Gliomas

**Figure 1:**
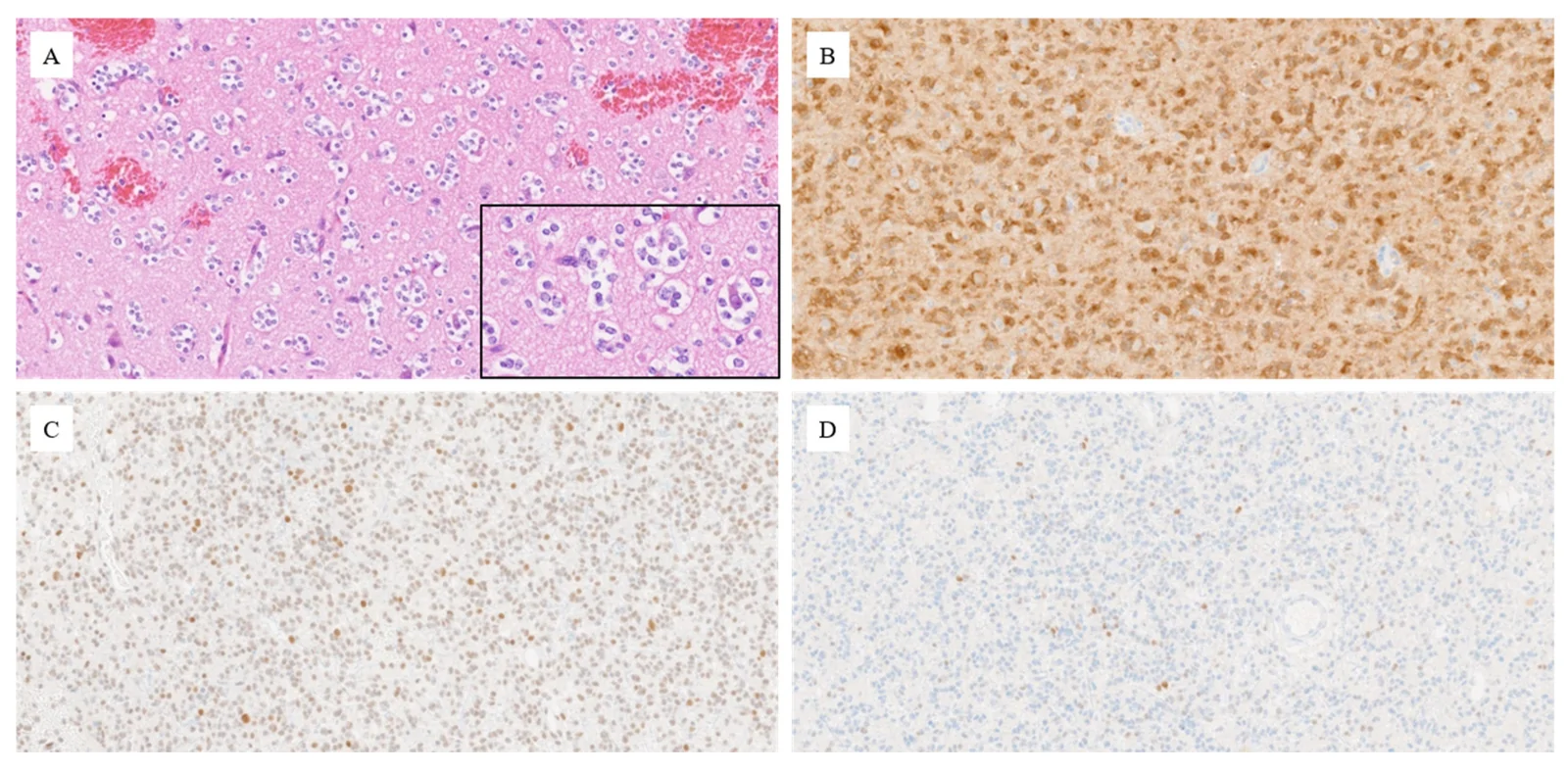
(**A**) Histopathology of IDH-mutant and 1p/19q-codeleted oligodendroglioma, showing a diffusely infiltrating glial tumor composed of a uniform population of cells with round nuclei and finely dispersed chromatin, exhibiting perinuclear clearing (“fried-egg” appearance), containing a delicate branching capillary network (“chicken-wire” vasculature). (**B**–**D**) Immunohistochemically, neoplastic cells are diffusely and strongly stained with IDH1 R132H (**B**) and show retained ATRX expression (**C**), while they lack diffuse p53 expression (**D**). Original magnifications: 200×.

Oligodendroglioma, IDH-mutant and 1p/19q-codeleted, remains the archetype of the phenotype herein discussed. Typical histology includes uniform round nuclei, clear cytoplasm, delicate branching “chicken-wire” vasculature, microcysts, and calcifications (Figure 1A). However, definitive diagnosis requires the demonstration of both an IDH1/2 mutation (Figure 1B) and whole-arm 1p/19q codeletion [1,2,3,4]. ATRX retention (Figure 1C) and absence of strong and diffuse p53 expression (Figure 1D) support this diagnosis, but they do not replace molecular confirmation of 1p/19q codeletion.

IDH-mutant astrocytoma may show focal oligodendroglioma-like areas, especially in higher-grade tumors. In contrast to oligodendroglioma, these tumors usually show ATRX loss and strong diffuse p53 expression, reflecting *ATRX* and *TP53* alteration [1,2]. Homozygous deletion of *CDKN2A/B* defines CNS WHO grade 4 in IDH-mutant astrocytoma, even in the absence of classic histological grade 4 features (i.e., necrosis and/or microvascular proliferation) [1]. The former category of oligoastrocytoma has largely disappeared because molecular classification has shown that most mixed-appearing tumors segregate into either astrocytoma or oligodendroglioma genotypes [2,8].

**Figure 2:**
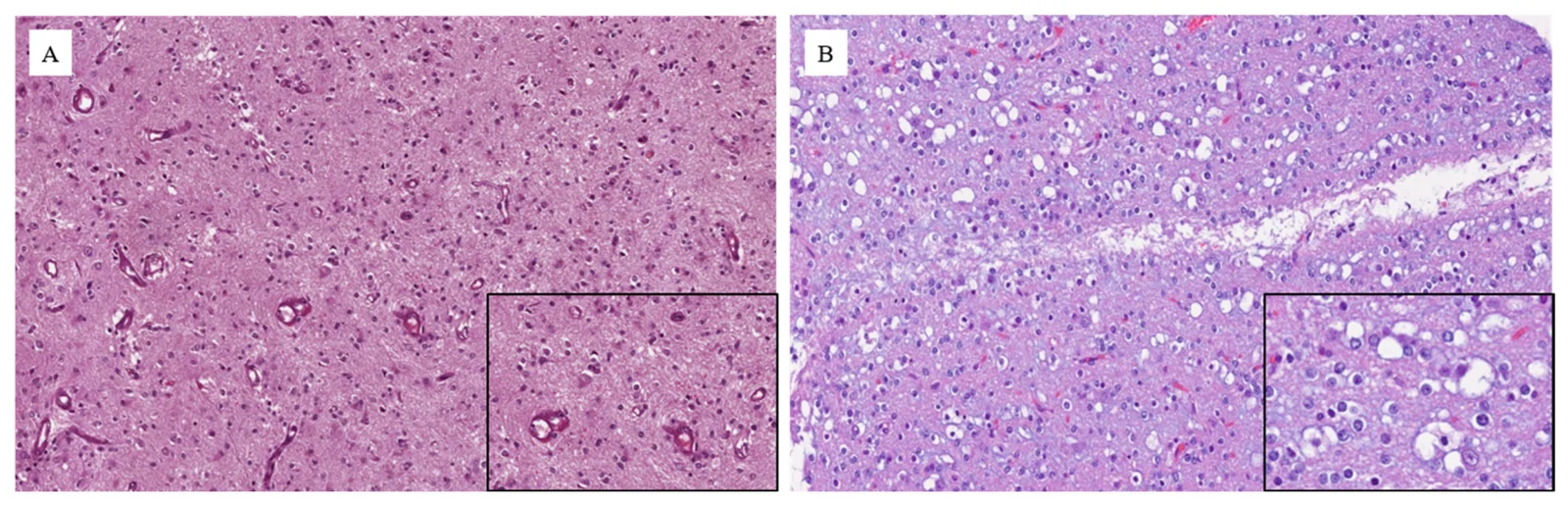
(**A**) Histologically, glioblastoma, IDH-wildtype with FGFR3::TACC3 fusion, H&E shows areas of lowly cellular glioma composed of monomorphic cells with round nuclei, high nuclear-to-cytoplasmic ratio, and perinuclear clearing, resulting in an overall oligodendroglioma-like appearance. A delicate branching capillary network is also seen. (**B**) DNT is composed of small oligodendrocyte-like cells arranged within mucin-rich matrix. A characteristic feature is the presence of “floating neurons” embedded in the mucinous background, reflecting its distinctive glioneuronal architecture. Original magnifications: 200×.

IDH-wildtype glioblastoma may represent a particularly treacherous mimic when it displays small-cell or oligodendroglioma-like morphology. Glioblastomas harboring *FGFR3::TACC3* fusion can show oligodendroglioma-like morphology with monomorphic cytology, compact growth, and limited necrosis in small samples, thereby mimicking a lower-grade diffuse glioma (Figure 2A) [5]. The mutual exclusivity between *FGFR3::TACC3* and *EGFR* amplification further complicates the differential diagnosis. In these settings, diffuse immunohistochemical expression of CD34 and some molecular criteria, such as the absence of IDH1/2 mutations, along with the detection of typical molecular features of glioblastoma (i.e., TERT-promoter mutation and/or combined chromosome 7 gain and chromosome 10 loss), may help to establish the diagnosis of glioblastoma and IDH-wildtype, and to consider the possibility of an *FGFR3::TACC3*-fusion, even when histology is deceptively bland [1,5,6,7].

### 3.2. Ependymal Tumors

Clear-cell morphology may occur in ependymomas and can closely simulate oligodendroglioma, particularly in supratentorial ependymomas with ZFTA fusion, while it is more unusual in YAP1-fused subgroup. Ependymomas with clear-cell (oligodendroglioma-like) morphology may show compact growth, uniform polygonal cells, and only subtle or absent perivascular pseudorosettes [14]. In a supratentorial glioma with partially circumscribed growth-pattern and perivascular arrangement with pseudorosettes, OLIG-2 negativity, along with EMA dot-like or ring-like positivity, supports ependymal differentiation. In supratentorial ependymomas, L1CAM expression and nuclear p65 immunoreactivity may support *ZFTA* fusion, whereas *YAP1*-fused tumors more often occur in younger children and generally show a more favorable course [14,15,16].

This group illustrates a key diagnostic principle: a cortical or frontal lesion with oligodendrocyte-like cells in a young patient is not automatically an oligodendroglioma. Ependymal differentiation must be actively excluded when morphology, location, or immunophenotype are discordant.

### 3.3. Pediatric-Type Low-Grade and Glioneuronal Tumors

Several pediatric-type low-grade and glioneuronal tumors may feature oligodendrocyte-like cells. These lesions are especially common in the setting of long-term epilepsy-associated tumors, where clinical presentation, cortical location, and radiological circumscription are essential diagnostic clues [17].

Although pilocytic astrocytoma (PA) classically demonstrates a conventional biphasic architecture with Rosenthal fibers and eosinophilic granular bodies, rare cases may exhibit extensive oligodendroglioma-like morphology [17]. In such cases, the distinction from “true” oligodendroglioma may be challenging, particularly in limited biopsy samples, and must be based on a careful evaluation of additional histological features (i.e., circumscribed growth pattern, biphasic architecture with Rosenthal fibers, and eosinophilic granular bodies), together with immunohistochemical and molecular studies. In contrast to oligodendroglioma, PA is characterized by activation of the MAPK pathway, most commonly through a *KIAA1549::BRAF* fusion or, less frequently, a *BRAF* p.V600E mutation, while lacking *IDH1/2* mutations and 1p/19q codeletion.

Dysembryoplastic neuroepithelial tumor (DNT) is an epilepsy-associated tumor, histologically characterized by oligodendrocyte-like cells arranged within a mucin-rich matrix and associated with floating neurons (Figure 2B). It is IDH-wildtype, is typically ATRX retained, and is frequently driven by *FGFR1* alterations [17,18,19]. Polymorphous low-grade neuroepithelial tumor of the young may contain scattered oligodendrocyte-like cells, microcystic architecture, calcifications, and diffuse CD34 expression, with recurrent MAPK pathway alterations including *FGFR2::CTNNA3* fusion or *BRAF* p.V600E mutation [9,10,11]. Angiocentric glioma may show oligodendroglioma-like areas, but its perivascular architecture and molecular profile support a distinct diagnosis [13].

**Figure 3:**
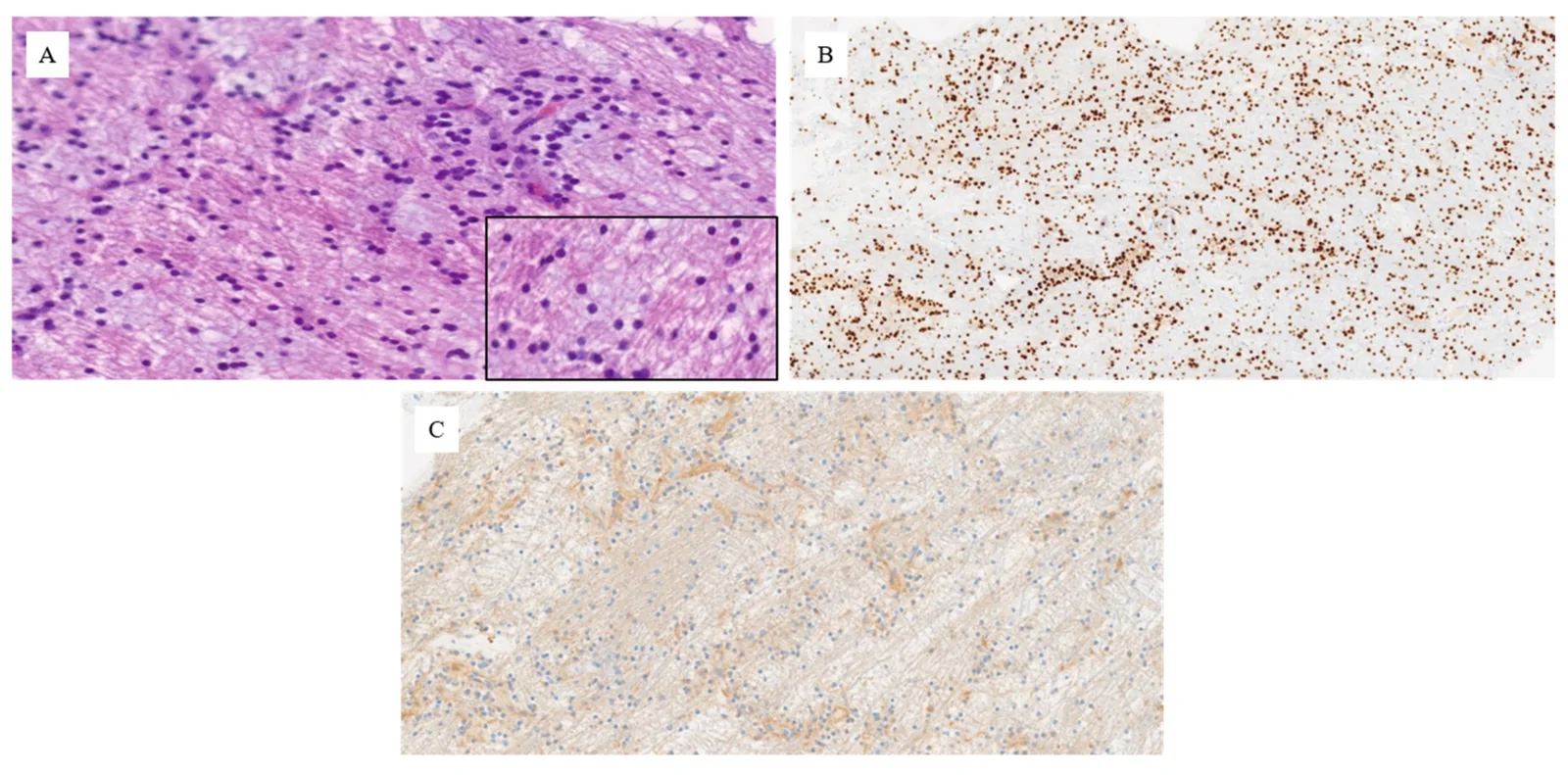
(**A**) Histology of myxoid glioneuronal tumor shows an oligodendroglioma-like pattern with round nuclei and focal perinuclear clearing within a monotonous cellular proliferation embedded in a myxoid matrix. (**B**,**C**) Neoplastic cells show diffuse nuclear immunoreactivity for OLIG2 (**B**) and Synaptophysin (**C**). Original magnifications: 200×.

Diffuse glioneuronal tumor with oligodendroglioma-like features and nuclear clusters is defined by a distinct DNA methylation profile, and is characterized by glioneuronal differentiation, nuclear clusters, presence of multinucleated cells, and absence of IDH1/2 mutation, along with recurrent monosomy 14 [20,21,22]. Diffuse leptomeningeal glioneuronal tumor may show oligodendrocyte-like cells, but the presence of leptomeningeal dissemination, *KIAA1549::BRAF* fusion, 1p loss, and, in more aggressive molecular subclasses, 1q gain [23,24], and lack of IDH1/2 mutations are relevant diagnostic criteria in the distinction from oligodendroglioma. Myxoid glioneuronal tumor is a low-grade neoplasm exhibiting small, rounded neurocytic cells embedded in a myxoid matrix (Figure 3A–C), and defined by *PDGFRA* p.K385 mutation, in which its typical anatomic location (i.e., septum pellucidum or periventricular region) represents a crucial diagnostic criterion [25].

**Figure 4:**
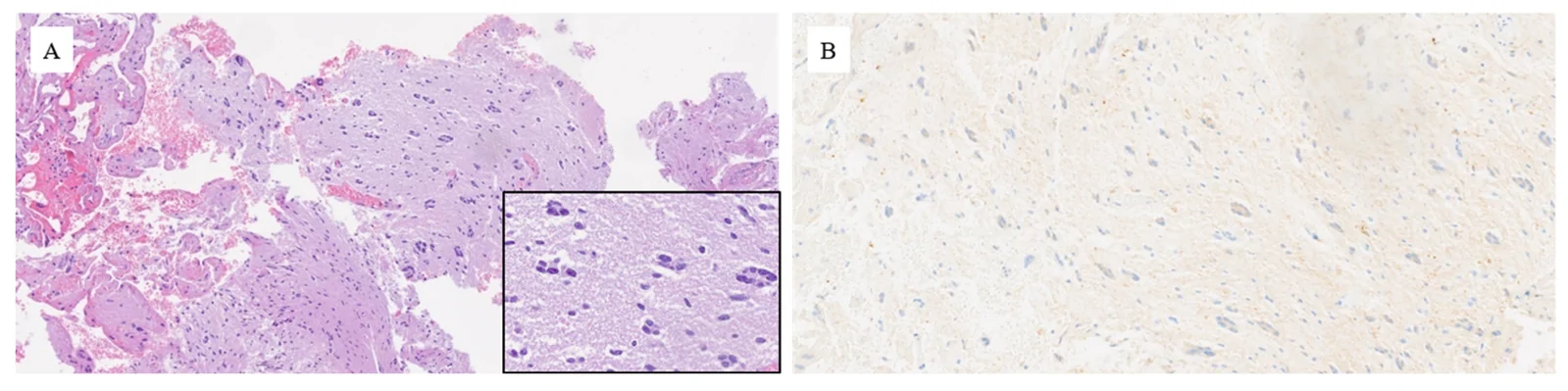
(**A**) Rosette-forming glioneuronal tumor is composed of cytologically bland neurocytic cells with true rosette formation. (**B**) Synaptophysin shows accentuated immunoreactivity within rosette lumina. Original magnifications: 200×.

Rosette-forming glioneuronal tumor (Figure 4A,B), papillary glioneuronal tumor, and multinodular and vacuolating neuronal tumor may also enter the differential diagnosis when focal clear-cell or oligodendroglioma-like morphology is present, but their architecture, location, and molecular alterations usually provide decisive clues [26,27,28].

### 3.4. Neurocytic Tumors

**Figure 5:**
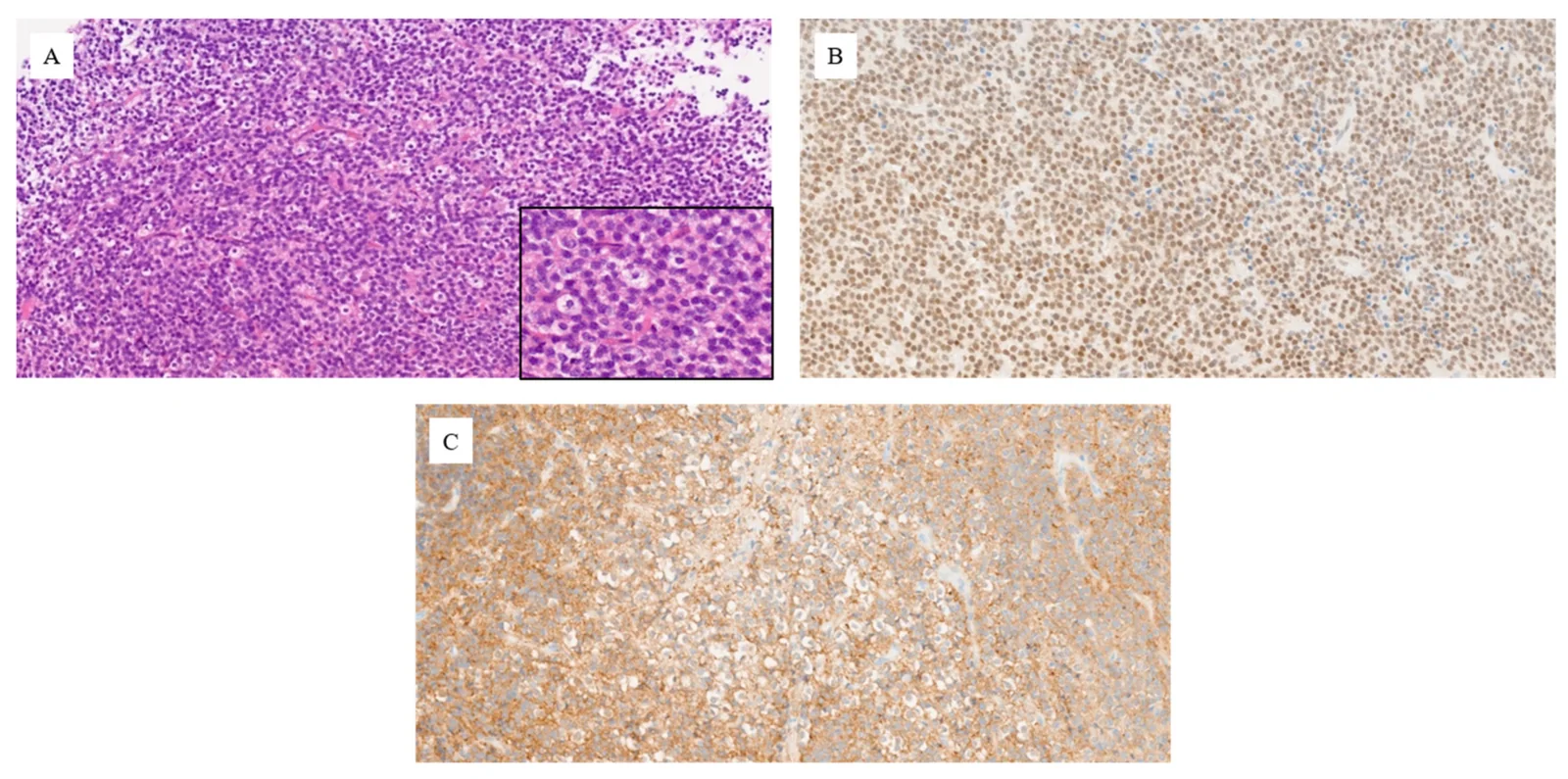
(**A**) Histopathology of central neurocytoma exhibits monomorphic neurocytic cells with round nuclei and delicate chromatin with perinuclear halo producing “fried-egg” oligodendroglioma-like morphology. (**B**,**C**) Diffuse immunohistochemical expression of NeuN (**B**) and Synaptophysin (**C**) are seen. Original magnifications: 200×.

Central neurocytoma and extraventricular neurocytoma may closely resemble oligodendroglioma because of uniform round nuclei of neurocytic cells, which also feature perinuclear halos (Figure 5A). However, the presence of neuropil islands, along with diffuse NeuN (Figure 5B) and Synaptophysin (Figure 5C) expression; absence of *IDH* mutation; and lack of 1p/19q codeletion are essential for distinction [29,30,31].

**Figure 6:**
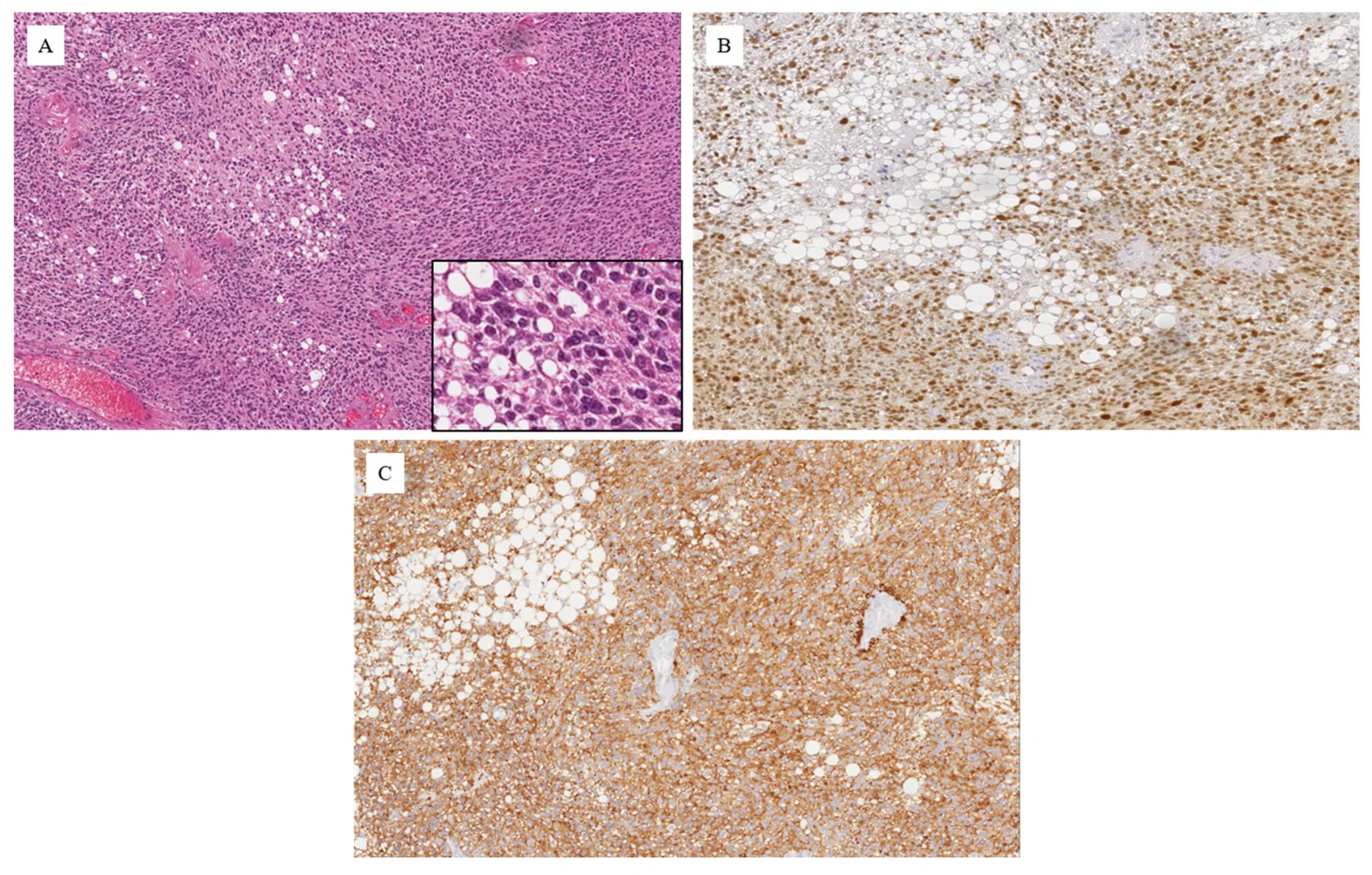
(**A**) Cerebellar liponeurocytoma is composed of uniform, rounded cells with focal clear-cell change and scattered lipidized elements. (**B**,**C**) Neuronal differentiation is confirmed by diffuse immunohistochemical expression of NeuN (**B**) and Synaptophysin (**C**). Original magnifications: 150×.

Cerebellar liponeurocytoma is another rare neurocytic tumor that often contains clear or lipidized cells and shows biphenotypic neuronal and glial differentiation (Figure 6A–C). Although generally indolent, it has potential for late recurrence and should not be mistaken for an oligodendroglioma [32].

### 3.5. Other Neoplastic Mimics with Clear-Cell Morphology

Metastatic renal cell carcinoma and hemangioblastoma may also enter in the differential diagnosis of clear-cell CNS lesions. Metastatic renal cell carcinoma may closely mimic primary clear-cell CNS tumors and requires epithelial and renal lineage markers such as cytokeratins, PAX8, and CAIX [38].

**Figure 7:**
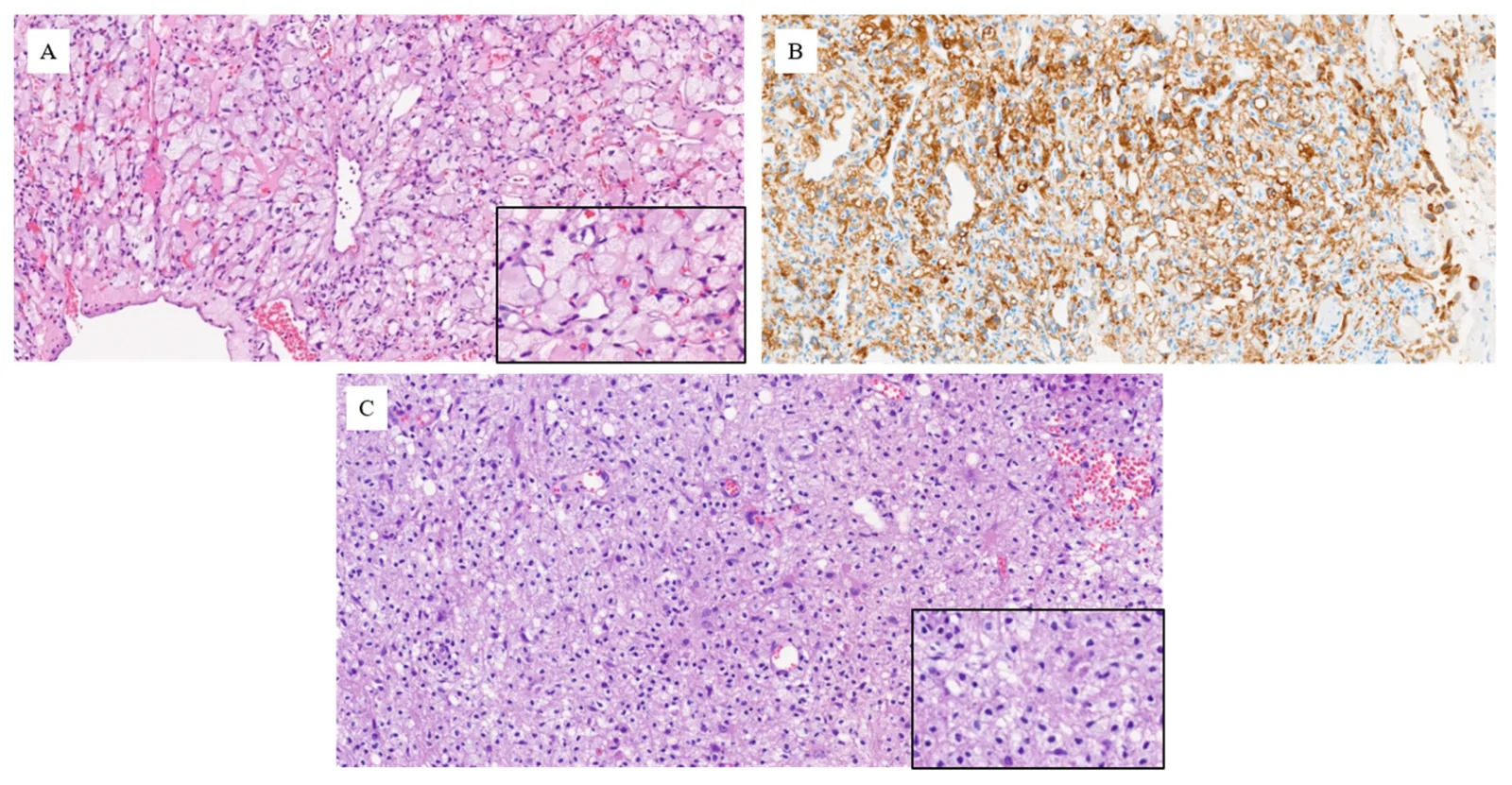
(**A**) Hemangioblastoma is composed of clear stromal cells combined with a dense arborizing capillary network. (**B**) Stromal cells show diffuse cytoplasmatic immunoreactivity for alpha-inhibin, supporting the diagnosis and aiding distinction from histological mimics. (**C**) H&E showing a demyelinating plaque characterized by perivascular and parenchymal inflammatory infiltrate, predominantly composed of foamy macrophages and lymphocytes. Original magnifications: 200×.

Hemangioblastoma combines stromal clear cells with a prominent vascular network (Figure 7A) and may be confused with metastatic renal cell carcinoma or other clear-cell lesions. Inhibin alpha (Figure 7B); S100; vascular markers; and clinical correlation, including possible von Hippel–Lindau disease, assist in classification [36,37].

### 3.6. Non-Neoplastic Mimics

Non-neoplastic lesions represent one of the most important sources of misdiagnosis. Subacute infarcts, tumefactive demyelinating lesions (Figure 7C), and inflammatory conditions may contain macrophage-rich areas, reactive oligodendroglial hyperplasia, vacuolated cells, or fixation artifacts that simulate low grade glioma [39,40,41]. Open-ring enhancement, incomplete mass effect, rapid radiological evolution, or a clinical context compatible with demyelination or ischemia should prompt caution.

CD68 and CD163 staining can highlight macrophage-rich inflammatory or ischemic lesions and prevent misinterpretation as neoplastic clear-cell populations. This point is clinically crucial because overcalling a non-neoplastic mimic as glioma may expose the patient to unnecessary surgery, radiotherapy, or chemotherapy.

## 4. Practical Integrated Diagnostic Workflow

**Figure 8:**
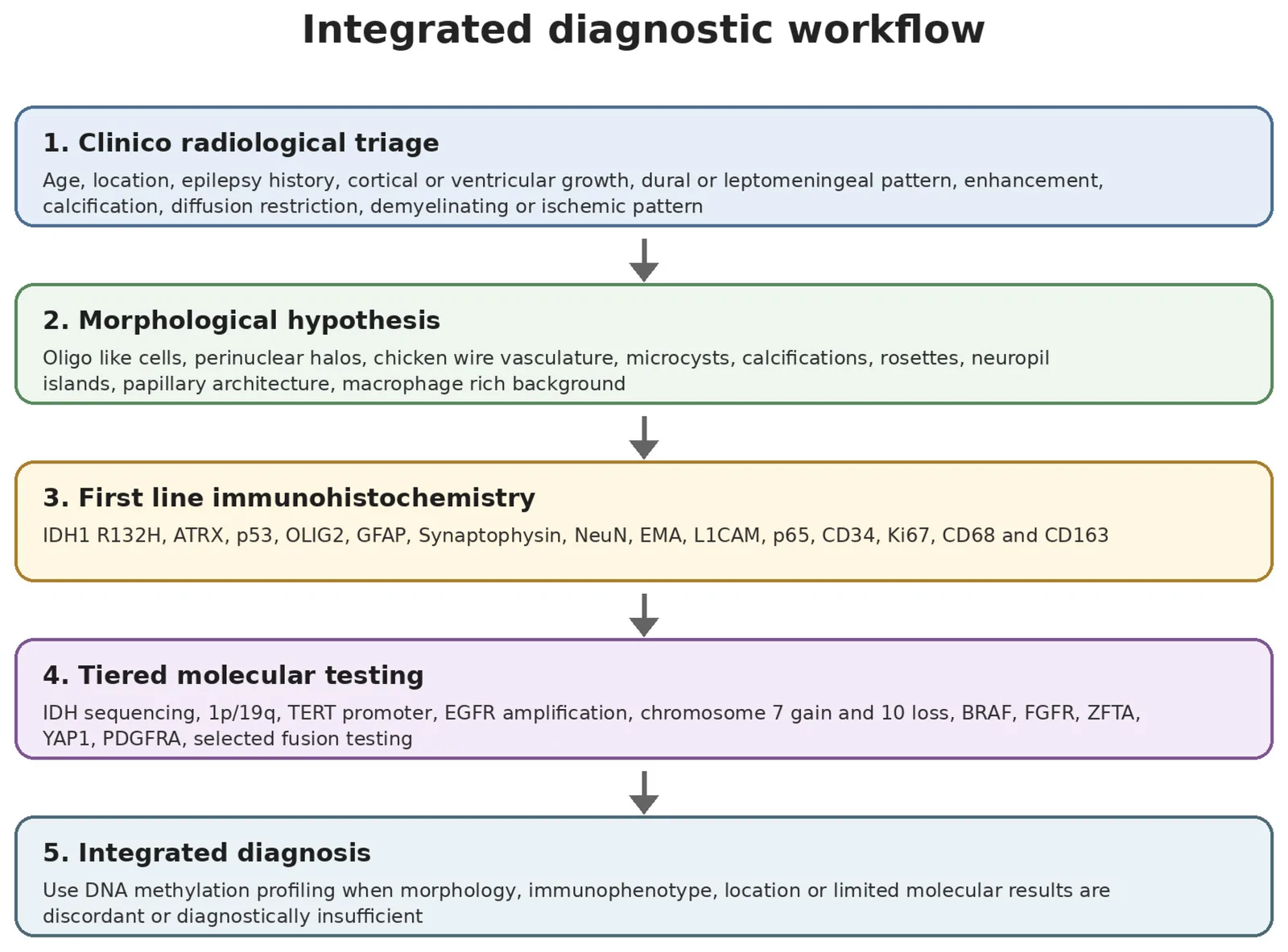
Practical diagnostic algorithm for CNS lesions with oligodendroglioma-like or clear-cell morphology.

A practical diagnostic approach begins with the recognition that morphology establishes a hypothesis, not a conclusion. The diagnostic sequence should proceed from clinical and radiological triage to morphology, immunohistochemistry, tiered molecular testing, and, when necessary, DNA methylation profiling (Table 2). Figure 8 summarizes a proposed workflow emphasizing that the oligodendroglioma-like pattern should trigger sequential interpretation rather than immediate classification. DNA methylation profiling is positioned as a problem-solving tool when the conventional diagnostic layers remain discordant or insufficient [42].

### 4.1. Clinico-Radiological Triage

Age and location often provide the first diagnostic filter. A cortical temporal lesion associated with long-standing epilepsy suggests a glioneuronal tumor more strongly than a diffuse adult-type glioma. An intraventricular lesion raises the possibility of central neurocytoma or ependymoma. A dural-based clear-cell lesion suggests meningioma or metastasis. A diffuse leptomeningeal process should prompt consideration of diffuse leptomeningeal glioneuronal tumor, infection, or inflammatory disease.

MRI features can further refine the differential diagnosis. T2 FLAIR mismatch favors IDH-mutant astrocytoma in the appropriate setting. A multinodular or “soap bubble” cortical lesion supports DNT or related epilepsy-associated tumors. Open-ring enhancement may suggest tumefactive demyelination. Marked diffusion restriction should raise the possibility of hypercellular embryonal tumors. Calcification is common in oligodendroglioma but is not specific.

### 4.2. First-Line Immunohistochemistry

A minimal first-line immunohistochemical panel should be tailored to the differential diagnosis generated by age, site, and morphology. In most adult-type diffuse glioma cases, IDH1 R132H, ATRX, p53, OLIG2, and Ki67 are essential. In clear-cell or oligodendroglioma-like lesions with possible neuronal or glioneuronal differentiation, Synaptophysin, NeuN, MAP2, and CD34 are useful additions. If ependymoma is possible, EMA, L1CAM, and p65 should be included. In suspected non-neoplastic mimics, CD68 and CD163 can clarify the macrophage component. If metastasis or meningioma is in the differential diagnosis, cytokeratins, PAX8, CAIX, SSTR2A, and EMA should be selected according to context.

Immunohistochemistry should not be interpreted mechanically. ATRX retention supports oligodendroglioma only in the proper molecular setting, and p53 overexpression suggests astrocytoma but must be considered with morphology and IDH status. OLIG2 positivity is not exclusive to oligodendroglioma. Similarly, focal neuronal marker expression does not by itself establish a glioneuronal tumor.

### 4.3. Tiered Molecular Testing

Molecular testing should answer the most clinically relevant diagnostic question first. In an adult-type diffuse gliomas, the initial priority is to define IDH status and 1p/19q codeletion. If IDH is wildtype, testing for molecular features of glioblastoma, including TERT-promoter mutation, EGFR amplification, and combined chromosome 7 gain and chromosome 10 loss, becomes critical [1].

In pediatric-type or glioneuronal tumors, MAPK pathway alterations, *FGFR1* or *FGFR2* alterations, *BRAF* fusion or mutation, *PDGFRA* p.K385 mutation, and selected gene fusions may be more informative than anadult diffuse glioma panel. For clear-cell ependymal tumors, *ZFTA* fusion assessment or validated surrogate markers (i.e., L1CAM and p65) may be diagnostically crucial. The choice of tests should therefore follow the diagnostic problem rather than a fixed universal panel.

### 4.4. When DNA Methylation Profiling Becomes Essential

Genome-wide DNA methylation profiling has transformed CNS tumor diagnostics by identifying biologically coherent epigenetic classes across histologically overlapping entities [2,3,42]. It is particularly valuable when morphology, immunophenotype, anatomical site, and limited molecular data do not converge on a single diagnosis.

Practical indications include unusual patient age; atypical location; discordant immunohistochemistry; lack of expected molecular alterations; small biopsies with limited diagnostic architecture; suspected methylation-defined entities, such as DGONC; and tumors with ambiguous glioneuronal, neurocytic, or embryonal features. However, methylation results should still be interpreted within the broader clinico-pathological context. A methylation class is not a replacement for diagnostic reasoning; it is a high-resolution layer within an integrated diagnosis.

## 5. Discussion

### 5.1. Why a True Oligodendroglioma May Be Misdiagnosed

The main diagnostic risk of oligodendroglioma-like morphology is not semantic but clinical, because “true” oligodendrogliomas, IDH-mutant and 1p/19q-codeleted, may benefit from targeted therapies and often show a more indolent course. The main reason an oligodendroglioma fails to be recognized and correctly diagnosed is the absence of immunohistochemical positivity for IDH1 (R132H); this—combined with retained ATRX expression and the absence of p53 overexpression—can lead to a misdiagnosis of IDH-wildtype glioblastoma, particularly in high-grade lesions. Accordingly, we strongly recommend IDH1/2 gene sequencing in diffuse gliomas with oligodendrocyte-like cells, retained ATRX expression, and lack of p53 overexpression—across all age groups but particularly in young adults—before establishing a diagnosis of IDH-wildtype diffuse glioma.

### 5.2. Minimal Diagnostic Panel in Routine Practice

In routine practice, a rational minimum panel can prevent most major errors (Box 1). For adult-type diffuse gliomas, IDH1 R132H, ATRX, p53, OLIG2, and Ki67 should be considered first-line markers, followed by IDH sequencing and 1p/19q testing when indicated. If IDH-wildtype status is confirmed, molecular testing for glioblastoma-defining alterations should be prioritized.

Box 1 **Red flags against a conventional oligodendroglioma diagnosis.**

- Pediatric age or very young adult age without the expected adult diffuse glioma context.
- Intraventricular, dural-based, leptomeningeal, sharply circumscribed cortical, or predominantly periventricular location.
- Absence of *IDH* mutation or absence of whole-arm 1p/19q codeletion.
- ATRX loss or strong diffuse p53 expression, suggesting IDH-mutant astrocytoma.
- EMA dot-like or ring-like staining, L1CAM positivity, or nuclear p65 expression, suggesting ependymal differentiation.
- Diffuse Synaptophysin, NeuN, or MAP2 expression with neuropil islands, suggesting neurocytic or glioneuronal lineage.
- Diffuse CD34 staining in a low-grade epilepsy-associated cortical tumor.
- Prominent CD68- or CD163-positive macrophage population, especially with demyelinating or ischemic radiological features.
- Cytokeratin, PAX8, CAIX, SSTR2A, or CD45 positivity, suggesting metastatic, meningeal, or hematolymphoid disease.
- Any major discordance between morphology, imaging, immunophenotype, and initial molecular results.

For lesions in children, young adults, or epilepsy-associated cortical locations, the panel should expand toward neuronal and glioneuronal markers, including Synaptophysin, NeuN, MAP2, and CD34, with subsequent molecular evaluation of MAPK pathway alterations. For clear-cell lesions with possible ependymal differentiation, EMA, L1CAM, and p65 are practical first-line tools. For macrophage-rich or radiologically atypical lesions, CD68 and CD163 are essential safeguards against misdiagnosis.

In a recent AOSNP-ADAPTR consensus, the authors proposed a resource-stratified diagnostic framework for adult-type diffuse gliomas to facilitate the implementation of the 2021 WHO CNS classification in laboratories with varying access to molecular testing [43]. The recommendations advocate a stepwise approach integrating histopathology, immunohistochemistry, and targeted molecular analyses according to available resources, highlighting the central role of *IDH* status, 1p/19q codeletion, *CDKN2A/B* homozygous deletion, and molecular markers of *IDH*-wildtype glioblastoma, and promoting transparent reporting when complete molecular profiling is unavailable [43].

### 5.3. Molecular Diagnostics and Methylation Profiling

Molecular diagnostics should be tiered according to diagnostic probability. A broad molecular panel may be ideal, but in many centers, it is neither immediately available nor cost-neutral. The practical alternative is a staged approach in which morphology and immunohistochemistry define the next most informative test. This strategy is especially important in resource-variable settings.

DNA methylation profiling is increasingly important when conventional workup remains unresolved. Its greatest strength is the ability to classify histologically overlapping tumors into biologically coherent entities and to reveal unexpected diagnoses. Its limitation is that it requires adequate tissue, appropriate quality control, and expert clinico-pathological interpretation. The most robust diagnoses arise when methylation class, copy number profile, histology, and clinical setting are mutually coherent [2,3,42].

### 5.4. Practical Implications

The integrated approach proposed here supports a shift from descriptive morphology to predictive morphomolecular diagnosis. In daily practice, this means that the pathologist should avoid ending the report at the level of “oligodendroglioma-like tumor” unless the case is explicitly unresolved and further testing is pending. Instead, the report should communicate the level of diagnostic certainty, the key supporting and discordant findings, and the specific ancillary tests required to reach final classification.

This approach is also relevant for multidisciplinary discussion. Radiologists can identify patterns that argue against conventional oligodendroglioma; neurosurgeons can provide information about sampling and lesion boundaries; and oncologists need an integrated diagnosis to select surveillance, radiotherapy, chemotherapy, or targeted strategies. The final diagnostic product is therefore not a microscopic label but a clinically actionable synthesis.

## 6. Conclusions

Oligodendroglioma-like morphology remains one of the most recognizable patterns in CNS pathology, but it is not synonymous with oligodendroglioma. In the current WHO era, its correct interpretation requires integration of morphology with clinical setting, imaging, immunohistochemistry, targeted molecular testing, and, when necessary, DNA methylation profiling. The essential diagnostic question is not simply whether a lesion resembles oligodendroglioma, but which biological entity is imitating it. Answering that question is the basis of accurate prognostic stratification and precision neuropathology.

## Figures and Tables

**Table 1:** Main neoplastic and non-neoplastic entities with oligodendroglioma-like or clear-cell morphology.

Entity or Group	Main Morphological Clues	Useful Immunohistochemistry	Key Molecular or Diagnostic Features	Practical Pitfall
Oligodendroglioma, IDH-mutant and 1p/19q codeleted	Uniform, round nuclei; perinuclear halos; chicken-wire vessels; microcysts; and calcifications.	IDH1 R132H-positive in most cases, ATRX retained, p53 not strong diffuse, and OLIG2-positive.	IDH1/2 mutation and whole-arm 1p/19q codeletion. Frequent TERT-promoter mutation.	Do not diagnose from morphology alone. 1p/19q codeletion is mandatory.
Astrocytoma, IDH-mutant	Focal oligodendroglioma-like areas may occur. More irregular nuclei and fibrillary or gemistocytic background may be present.	IDH1 R132H-positive, ATRX loss, strong diffuse p53, and OLIG2-positive.	IDH mutation without 1p/19q codeletion. CDKN2A/B homozygous deletion defines grade 4.	May be mistaken for oligodendroglioma if oligodendrocyte-like cells are diffusely present.
Glioblastoma, IDH-wildtype	Small-cell or oligodendroglioma-like morphology, compact growth, and deceptively bland cytology in limited biopsies.	IDH1 R132H-negative, variable p53, high Ki67, and often OLIG2-positive.	TERT-promoter mutation, EGFR amplification, chromosome 7 gain and 10 loss, and possible FGFR3::TACC3 fusion (if present, mutually exclusive with EGFR amplification).	May look lower grade if necrosis or microvascular proliferation is absent in the sample.
Ependymoma	Uniform round cells, compact architecture, and subtle or absent pseudorosettes.	EMA dot or ring pattern, L1CAM-positive and nuclear p65 in ZFTA-fused tumors, and usually OLIG2-negative.	ZFTA fusion or YAP1 fusion in supratentorial ependymomas (clear-cell morphology primarily associated with ZFTA-fused tumors, while unusual in YAP1-fused subgroup).	Can closely mimic cortical oligodendroglioma.
DNT and other epilepsy-associated glioneuronal tumors	Oligodendrocyte-like cells, mucin-rich matrix, floating neurons, and multinodular cortical architecture.	Synaptophysin highlights neuronal component, OLIG2 variable, and CD34 in selected entities.	Frequent FGFR1 or MAPK pathway alterations; IDH-wildtype.	Clinical epilepsy history and cortical location are often decisive.
DGONC	Oligodendroglioma-like areas with nuclear clusters and glioneuronal differentiation.	OLIG2 and neuronal marker co-expression may be present.	Methylation-defined entity; recurrent monosomy 14.	Often requires methylation profiling for confident classification.
DLGNT	Oligodendrocyte-like cells with leptomeningeal dissemination.	Glial and neuronal marker expression.	KIAA1549::BRAF fusion, 1p loss, and sometimes 1q gain.	Leptomeningeal pattern should redirect the differential diagnosis.
Central or extraventricular neurocytoma	Uniform neurocytic cells, perinuclear halos, and neuropil islands.	Diffuse Synaptophysin and NeuN, MAP2-positive, and GFAP limited to reactive astrocytes.	No single recurrent driver. Methylation profiling may refine classification.	Intraventricular location and neuronal markers argue against oligodendroglioma.
Clear-cell meningioma	Glycogen-rich clear cells arranged in sheets, deposition of interstitial and perivascular thick amianthoid-like collagen, and possible dural relationship.	EMA- and SSTR2A-positive, and glial- and neuronal-markers-negative.	SMARCE1 loss in a subset.	May lack obvious dural attachment.
Metastatic renal cell carcinoma	Clear cells, vascular network, and epithelial architecture may be subtle.	Cytokeratin-, PAX8- and CAIX-positive.	Metastatic carcinoma diagnosis supported by systemic workup.	Can mimic hemangioblastoma or primary clear-cell CNS tumors.
Hemangioblastoma	Clear stromal cells and prominent vascular network.	Inhibin alpha and S100 in stromal cells, and vascular markers in capillary network.	Consider von Hippel–Lindau context.	May resemble metastatic renal cell carcinoma.
Tumefactive demyelination or subacute infarct	Macrophage-rich lesion, reactive gliosis, vacuolated cells, or oligodendroglial hyperplasia.	CD68- and CD163-positive macrophages; myelin stains helpful.	Non-neoplastic diagnosis supported by imaging and clinical evolution.	Major source of overdiagnosis as low-grade glioma.

**Table 2:** Practical tiered workup for oligodendroglioma-like or clear-cell CNS lesions.

Diagnostic Layer	Main Question	Suggested Tools	How the Result Should Be Used
Clinical and radiological triage	Does age, site, or imaging fit conventional oligodendroglioma?	Age, seizure history, lesion site, enhancement pattern, diffusion restriction, calcification, and leptomeningeal or dural pattern.	Defines the initial differential diagnosis and identifies red flags.
Histology	Is the oligodendroglioma-like pattern pure or associated with another architecture?	Search for chicken-wire vessels, microcysts, calcifications, rosettes, neuropil islands, papillae, macrophage-rich areas, necrosis, and microvascular proliferation.	Generates the morphological hypothesis and selects the immunohistochemical panel.
First-line diffuse glioma panel	Is this an adult-type diffuse glioma?	IDH1 R132H, ATRX, p53, OLIG2, GFAP, and Ki67.	Separates likely oligodendroglioma, astrocytoma, and IDH-wildtype glioma patterns.
Lineage expansion panel	Is there ependymal, neuronal, meningeal, metastatic, or lymphoid differentiation?	EMA, L1CAM, p65, Synaptophysin, NeuN, MAP2, CD34, SSTR2A, cytokeratins, PAX8, CAIX, CD45, CD20, CD68, and CD163.	Redirects diagnosis when the oligodendroglioma-like pattern is a mimic.
Molecular confirmation	Which molecular alteration defines the entity?	IDH sequencing; 1p/19q testing; TERT promoter; EGFR amplification; chromosome 7 and 10 copy number; and BRAF, FGFR, PDGFRA, ZFTA, and YAP1 fusion testing, if indicated.	Confirms integrated diagnosis and prevents morphology driven misclassification.
DNA methylation profiling	Do conventional layers remain discordant or insufficient?	Genome-wide methylation array with copy number profile and classifier score.	Useful for unusual age, atypical site, ambiguous glioneuronal or neurocytic tumors, small biopsies, and suspected methylation-defined entities.
Final integrated report	Can the diagnosis be stated with biological coherence?	Synthesis of histology, IHC, molecular data, radiology, and clinical setting.	Reports final entity, grade when applicable, pending tests, and diagnostic limitations.

## Data Availability

No new data were created or analyzed in this study. Data sharing is not applicable to this article.
